# Plant poisoning leads to alpha-synucleinopathy and neuromelanopathy in kangaroos

**DOI:** 10.1038/s41598-019-53396-8

**Published:** 2019-11-13

**Authors:** Mourad Tayebi, Charles M. El-Hage, Pedro Pinczowski, Pam Whiteley, Monique David, Qiao-Xin Li, Shiji Varghese, Meena Mikhael, Umma Habiba, David Harman, Liliana Tatarczuch, Mirjana Bogeski, Ian Birchall, Kirsty Ferguson, Larry Walker, Colin Masters, Brian A. Summers

**Affiliations:** 1 School of Medicine, Western Sydney University, Campbelltown, NSW Australia; 2 University of Melbourne, Parkville, Victoria Australia; 3 New South Wales Department of Primary Industries, Menangle, New South Wales Australia; 4 Florey Institute of Neuroscience and Mental Health, Parkville, Victoria Australia; 5 MGV Mt Annan, Mt Annan, NSW Australia; 6 Sydney Metropolitan Wildlife Services, Lindfield, NSW Australia; 7 Southern Scientific, Hamilton, Victoria Australia; 8 Emeritus Professor, Frankston, Victoria Australia

**Keywords:** Neurodegeneration, Neurodegenerative diseases

## Abstract

The pathogenesis of synucleinopathies, common neuropathological lesions normally associated with some human neurodegenerative disorders such as Parkinson’s disease, dementia with Lewy bodies and multiple system atrophy, remains poorly understood. In animals, ingestion of the tryptamine-alkaloid-rich phalaris pastures plants causes a disorder called Phalaris staggers, a neurological syndrome reported in kangaroos. The aim of the study was to characterise the clinical and neuropathological changes associated with spontaneous cases of Phalaris staggers in kangaroos. Gross, histological, ultrastructural and Immunohistochemical studies were performed to demonstrate neuronal accumulation of neuromelanin and aggregated α-synuclein. ELISA and mass spectrometry were used to detect serum-borne α-synuclein and tryptamine alkaloids respectively. We report that neurons in the central and enteric nervous systems of affected kangaroos display extensive accumulation of neuromelanin in the perikaryon without affecting neuronal morphology. Ultrastructural studies confirmed the typical structure of neuromelanin. While we demonstrated strong staining of α-synuclein, restricted to neurons, intracytoplasmic Lewy bodies inclusions were not observed. α-synuclein aggregates levels were shown to be lower in sera of the affected kangaroos compared to unaffected herd mate kangaroos. Finally, mass spectrometry failed to detect the alkaloid toxins in the sera derived from the affected kangaroos. Our preliminary findings warrant further investigation of Phalaris staggers in kangaroos, potentially a valuable large animal model for environmentally-acquired toxic synucleinopathy.

## Introduction

The molecular mechanisms underlying the accumulation of intracytoplasmic α-synuclein inclusions in human neurodegenerative disorders, including idiopathic Parkinson’s disease (PD), PD dementia, dementia with Lewy bodies (DLB) and multiple system atrophy (MSA)^1^ remains elusive. The demonstration that the nigrostriatal-toxic addictive ‘street designer drug’ N-methyl- 4-phenyl-l,2,3,6-tetrahydropyridine (MPTP), structurally analogous to tryptamine alkaloids (TA)-derivatives produced *in vivo*^2^, leads to long-lasting parkinsonism in humans and monkeys^3–6^, highlighted the possible involvement of alkaloids in Parkinsonism syndromes, including idiopathic Parkinson’s disease^7,8^. Furthermore, MPTP and MPTP analogues, shown to cause dopaminergic diminution in owl monkeys^9^ and C57/B1 mice^10^, failed to cause chronic depletion of nigrostriatal dopamine in α-synuclein knockout mice, suggesting that α-synuclein influences alkaloids neurotoxicity^11^.

Phalaris toxicity (PT) affects animals at pasture, most commonly sheep but cattle and horses can also be susceptible to the Phalaris-derived toxins following ingestion of various Phalaris spp such as *P. aquatica*^12–19^. PT has been reported since the 1940’s with a worldwide prevalence^12,17,20,21^ and occurs in grazing animals characteristically during a prolonged period of dry weather that precedes rainfall^22^. Because these are widely cultivated perennial pasture plants, over many decades phalaris toxicity in livestock has been important in Australia, New Zealand, South Africa, USA and Norway^23^. Outbreaks of poisoning occur worldwide at certain times of the year and are limited as far as possible by management procedures such as grazing on mature pastures which are less toxic than young plants.

PT can manifest as a ‘sudden death’ or as a ‘staggers’ form^24^; and the clinical presentation of the former is described as either neurologic or cardiac^22,24^. Staggers, which affect gait and locomotion of affected animals is further subdivided into an acute reversible or the more common lethal chronic form (also called Phalaris staggers)^24^. Sheep affected with Phalaris staggers display a more severe clinical disorder. Following introduction to Phalaris pastures, sheep initially display subtle neurological signs in the first three weeks^25–27^ progressively becoming more severe and pronounced after three to four months^12^. Neurological signs described include incoordination, hyper-excitability, muscle tremors, abnormal gait, thoracic and pelvic limb paresis, convulsions, recumbency, falling and death^18,26^. In cattle, Phalaris staggers leads to weakness, muscle tremors, ataxia and staggering associated with pelvic limb paresis leading to recumbency and death^23,25^.

Phalaris staggers affecting eastern grey kangaroos (*Macropus giganteus*) has been reported previously but its incidence remains unknown. However, it is believed to be high in certain Australian regions following exposure to Phalaris-rich pastures (El-Hage, unpublished work, 2017). Phalaris staggers was also reported in red kangaroos (*Macropus rufus*), wallabies (Munday, personal communication cited in^22^), western grey kangaroos (*Macropus fuliginosus fuliginosus*) and Tamar wallabies [(*M. eugenii;* Philips, unpublished work, 1984 cited in^22^)]. Bacci and colleagues described the syndrome in seven eastern grey kangaroos (*Macropus giganteus*) grazing Phalaris pastures^22^. The clinical presentation resembled that seen in sheep and included the typical Phalaris-associated neurological signs such as ataxia, hyper-excitability, muscle tremors, abnormal gait, paresis, convulsions, recumbency and death^22^. Furthermore, the neuropathological changes described in these kangaroos were reported to be similar to those described in sheep affected with Phalaris staggers and included pronounced green discoloration of the grey matter, that appeared microscopically as intraneuronal brown pigmentation^22^.

It has been proposed that Phalaris staggers is caused by tryptamine alkaloids (TA) found in Phalaris species [7, 17]; these alkaloids are structurally comparable to serotonin^27^, are serotonergic receptor agonists, and have a high affinity for serotonin receptors^28^. Following administration of TA to sheep, they developed clinical signs similar to Phalaris staggers, supporting an etiological role of these alkaloids in the pathogenesis of Phalaris toxicity^29^. Of note, failure of MPTP and MPTP analogues to deplete nigrostriatal dopamine in α-synuclein knockout mice^11^ may indicate that they also interact with Phalaris-derived alkaloids in kangaroos, sheep and cattle.

In this study, we describe the presence of α-synucleinopathy and melanopathy in the nervous system of Phalaris staggers-affected eastern grey kangaroos. Histology, immunohistochemistry (IHC) and electron microscopy (EM) were applied to confirm the presence of melanin pigments in the neurons of the central nervous system and the enteric nervous system of eastern grey kangaroos exposed to Phalaris pastures. Of importance, we also show and for the first time, the presence of intracytoplasmic α-synuclein inclusions in the neurons and sera of these kangaroos. Finally, using a sensitive UHPLC-MS/MS method, we were unable to detect tryptamine alkaloids in sera of Phalaris staggers-affected eastern grey kangaroos.

## Results

### Clinical presentation

All Phalaris-affected EGK displayed signs of a neurologic disorder which typically included ataxia, head shaking, erratic hopping and generalized muscle tremors. Upon perceived threats, episodes of pronounced hyperexcitability were also observed.

### Haematoxylin–eosin staining displays intra-neuronal pigmentation

**Figure 1:**
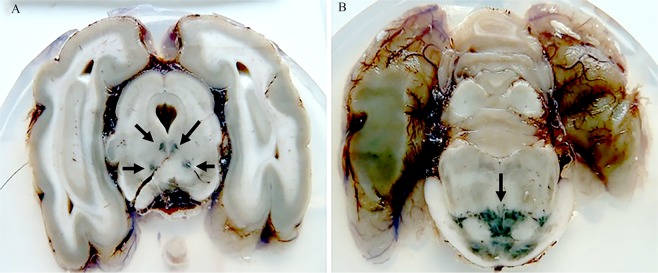
Macroscopic appearance of brain from a Phalaris-affected eastern grey kangaroo. Transverse section of the brain from case EGK89 displaying conspicuous bilateral and symmetrical, greenish, grey matter discoloration (arrows point to areas of pigmentation). (**A**) Shows the oculomotor nucleus and the red nucleus. Substantia nigra is evident but appears unaffected. (**B**) Shows the pontine nucleus. Representative of all affected Kangaroos.

Macroscopic examination of serial transverse sections of the brains and spinal cords from Phalaris-affected EGK revealed widespread and conspicuous gross greenish grey matter discoloration mainly affecting the cerebral cortex, thalamus, brainstem and spinal cord (Fig. 1). Control brains and spinal cords of the healthy unaffected EGK sourced from the archive collection did not display similar pigmentary abnormalities. This suggests a link between Phalaris grass ingestion in EGK and the gross anatomical changes because neuronal pigmentation is well documented in Phalaris staggers of affected sheep and other domestic animals^12,18,19,30,31^.

**Figure 2:**
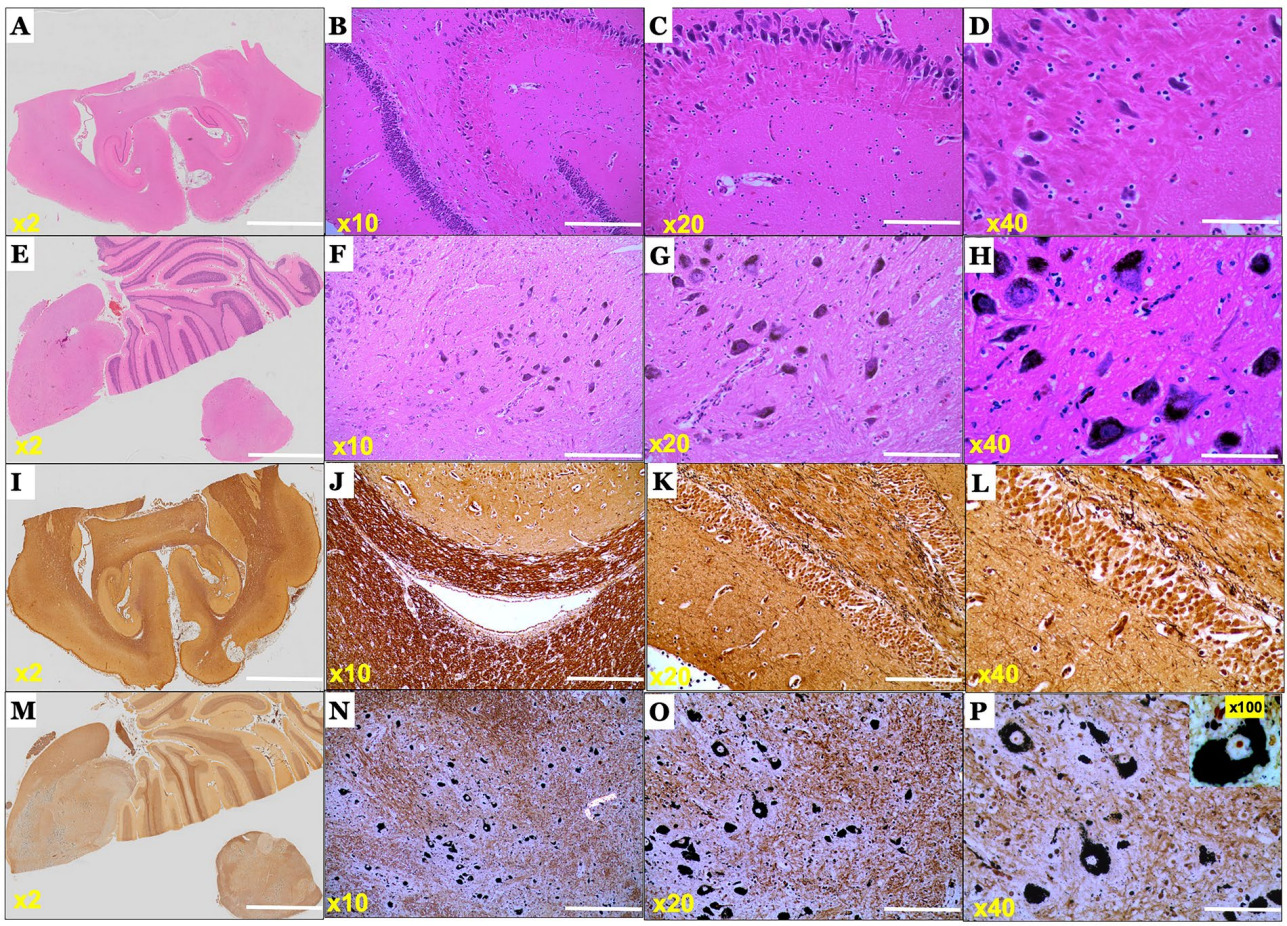
Photomicrographs of the microscopic lesions in the central nervous system of a Phalaris-affected eastern grey kangaroo. (**A**) Normal appearance of the brain parenchyma in the healthy EGK (case 15-11801). (**B–D**) Are higher magnification of (**A**). (**E**) Intense neuromelanin-like brown pigments in neurons observed on routine H&E stained sections of brain of EGK89. (**F–H**) Are higher magnification of (**E**). Representative of all affected Kangaroos. (**I**) Silver stain reaction (Warthin Starry) did not display the presence of neuromelanin in the healthy EGK case 15–11801. (**J–L**) Are higher magnification of (**I**). Note absence of pigments in neurons. (**M**) Extensive intracytoplasmic melanosis in cerebral cortex derived from case EGK89, revealed by Warthin Starry reaction stain. (**N–P**) Are higher magnification of (**K**). Representative of all affected Kangaroos.

**Figure 3:**
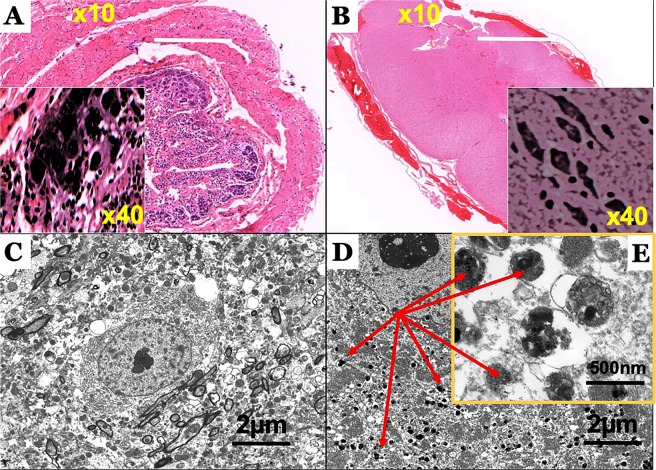
Photomicrographs of the microscopic and ultramicroscopic lesions in the spinal cord and enteric nervous system of a Phalaris-affected eastern grey kangaroo. (**A**) Intense neuromelanin-like brown pigments in neurons observed on routine H&E stained sections of intestine and (**B**) spinal cord from case EGK89. Representative of all affected Kangaroos. (**C**) Transmission electron microscopic (TEM) analysis of healthy EGK (case 15-11801) did not display electron-dense neuromelanin. (**D**) TEM analysis reveals intra-neuronal electron-dense neuromelanin (red arrows) in a Phalaris-affected EGK (EGK 92) with (**E**) lipid bulbs (arrow) attached to the granule.

We then performed haematoxylin–eosin (H&E) staining to assess the tissue morphology of brains (Fig. 2A–M), spinal cords (Fig. 3A) and gastro-intestinal tract (Fig. 3B) of Phalaris-affected and unaffected EGK. Sections of brain (n = 15), spinal cord (n = 10) and the intestinal tract (n = 5) from all Phalaris-affected EGK displayed light to intense neuromelanin-like brown pigments in neurons. These pigments appeared as conspicuous and uniformly sized granules scattered within the cytoplasm (Fig. 2H). Of note, and in contrast to previous reports^22^, the H&E stain revealed that accumulation of pigments in the cytoplasm of neurons did not displace the nuclei of affected neurons nor did it alter the integrity of their cell membranes. Of note, 5 areas of each region of the brain were analysed. The melanin-like pigmentation was consistently observed in the same anatomical subdivisions of all Phalaris-affected kangaroos, typically including midbrain and brainstem as well as the dorsal horns of the spinal cord. The microscopic presentation observed in phalaris-affected EGK was not seen in any of the unaffected EGK (Fig. 2M–P).

### Special histochemical stain displays intense intra-neuronal neuromelanin-like granules

Special staining to substantiate neuromelanin deposits was performed as per Orchard GE^32^. With the exception of the Warthin-Starry (WS) reaction, other stains such as Fontana-Masson and Schmorl’s stains were only able to faintly bind to the intraneuronal granules revealed by H&E in the brains and spinal cords of Phalaris-affected EGK (data not shown). For visualization of the melanin-like granules in Phalaris-affected EGK with WS reaction (Fig. 2), an aqueous silver nitrate solution in combination with hydroquinone reducing agent was applied to serial brain and spinal cord sections. WS stain relies on the ability of certain pigments, including melanin to bind silver ions from solution. Of importance, we elected to use WS to aid in the identification of the granules revealed by H&E stain due to its superior binding to melanin and neuromelanin compared to other suboptimal reactions, such as Fontana-Masson and Schmorl’s stains^33,34^. WS stain displayed intense dark granular staining in Phalaris-affected EGK sometimes leading to complete obliteration of the cell cytoplasm while retaining a clear interface with the neuropil (Fig. 2P). WS-specific neuromelanin was confined to cell neuronal compartments. Moreover, WS-specific melanin was not seen in the brains of unaffected kangaroos (Fig. 2G–I). Of note, no pigments were seen in the Purkinje cells of the cerebellum of Phalaris affected EGK; however, Golgi type II neurons were intensely stained (data not shown).

### Ultrastructure confirmation of the presence of intra-neuronal neuromelanin granules

Intra-neuronal granules that contain neuromelanin differ biologically and structurally from melanosomes which are normally found in skin and hair melanocytes and are responsible for melanin synthesis^35^. Transmission electron microscopic analysis of 5 Phalaris-affected EGK (EGK 92) and control (Fig. 3C,D) displayed the usual morphological ‘architecture’ typically associated with neuromelanin (Fig. 3D) and similar to those found in the dopaminergic neurons located in the substantia nigra pars compacta^36^. Here, neuromelanin was observed within neurons and deposited as a granular type with no fibrillar or vesiculoglobular matrix normally associated with melanin structures^37,38^. Furthermore, neuromelanin bodies displayed indistinct borders, were variable in size and presented as lobulated forms (15, 16). Moreover, neuromelanin was associated with what appears to be lipid bulbs; lipofuscin-filled vacuolar structures attached to neuromelanin containing-granules (Fig. 3D)^36^.

### Detection of α-synuclein in the brain and serum of Phalaris-affected EGK

**Figure 4:**
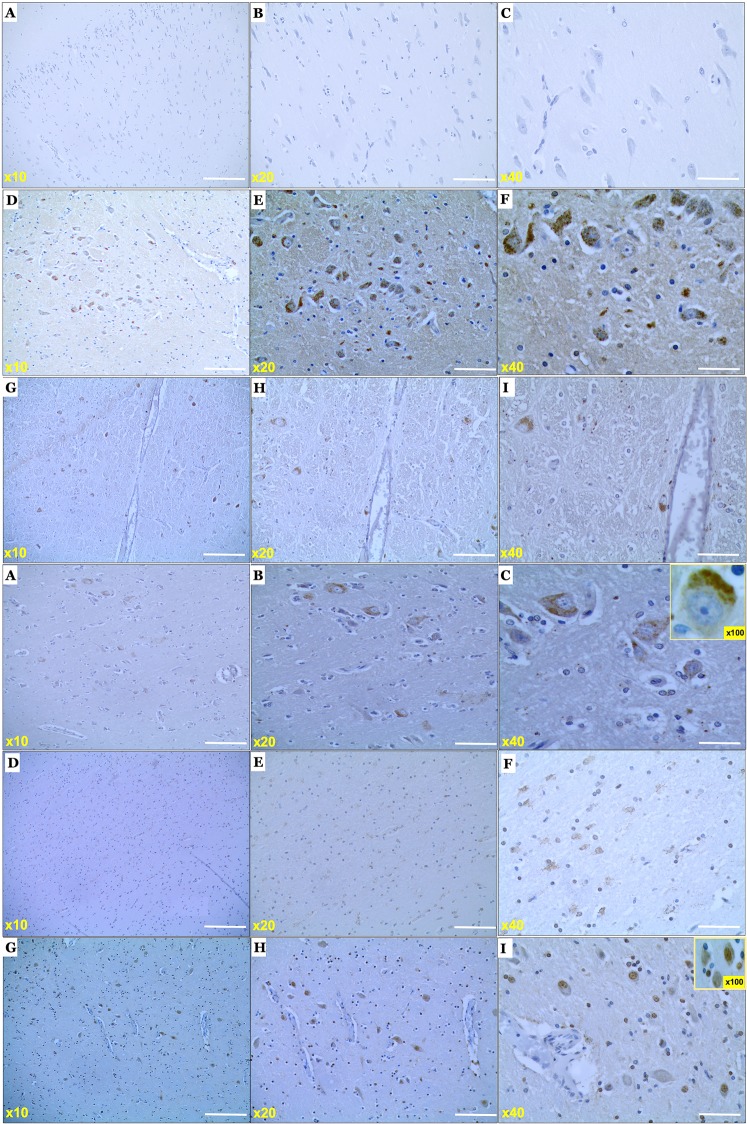
Photomicrographs of the α-synucleinopathy in the central nervous system of a Phalaris-affected eastern grey kangaroo. (**A**) Immunohistochemical staining of healthy EGK (case 15–11801) with rabbit anti-human α-synuclein polyclonal IgG [97/8; 1:2000 dilution]. (**B**,**C**) Are higher magnification of (**A**). (**D**) Immunohistochemical staining with rabbit anti-human α-synuclein polyclonal IgG [97/8; 1:2000 dilution] of a Phalaris-affected EGK (EGK 92) which shows multi-shaped aggregates ranging from ovoid and fusiform to threadlike intensely stained structures. (**E**,**F**) Are higher magnification of (**D**). (**G**) Immunohistochemical staining with rabbit anti-human α-synuclein polyclonal IgG [97/8; 1:2000 dilution] of a Phalaris-affected EGK (EGK 59) which shows multi-shaped aggregates ranging from ovoid and fusiform to threadlike intensely stained structures. (**H**,**I**) Are higher magnification of (**G**). (**J**) Immunohistochemical staining with rabbit anti-human α-synuclein polyclonal IgG [97/8; 1:2000 dilution] of a Phalaris-affected EGK (EGK 42) which shows multi-shaped aggregates ranging from ovoid and fusiform to threadlike intensely stained structures. (**K**,**L**) Are higher magnification of (**J**). (**M**) Immunohistochemical staining with rabbit anti-human α-synuclein polyclonal IgG [MJRF1; 1:2000 dilution] of a Phalaris-affected EGK (EGK 92) which shows multi-shaped aggregates ranging from ovoid and fusiform to threadlike intensely stained structures (**N**,**O**). Are higher magnification of (**M**). (**P**) Immunohistochemical staining with rabbit anti-human α-synuclein polyclonal IgG [MJRF1; 1:2000 dilution] of a Phalaris-affected EGK (EGK 59) which shows multi-shaped aggregates ranging from ovoid and fusiform to threadlike intensely stained structures. (**Q**,**R**) Are higher magnification of (**P**). Representative of all affected kangaroos.

**Figure 5:**
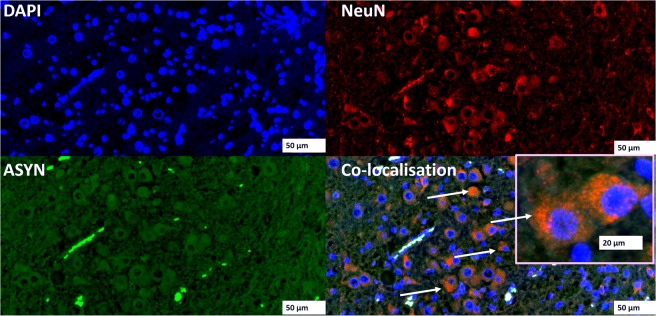
Immunofluorescence co-localisation of α-synuclein aggregates and neuron-specific nuclear protein, NeuN: Cortical co-staining with rabbit anti-human α-synuclein polyclonal IgG [97/8; 1:2000 dilution] (**GREEN**) and anti-mouse NeuN monoclonal IgG (Millipore, 1:2000 dilution) (**RED**) of phalaris-affected EGK92. DAPI (**BLUE**) was used to stain the nuclei. Representative of all affected kangaroos.

**Figure 6:**
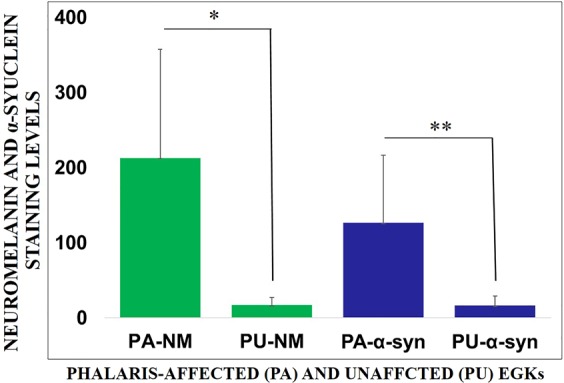
Quantitation of neuromelanin- and α-synuclein-laden neurons in Phalaris-affected and unaffected EGKs. Neuromelanin and α-synuclein staining levels of Phalaris-affected (n = 8) EGKs in cerebrum, mid-brain, cerebellum and spinal cord. The data are mean ± SEM from 5 randomly selected images per section. Similar regions from unaffected (n = 4) EGKs were used as a control. Neuromelanin and α-synuclein staining levels were quantified via ImageJ software as the relative intensity of neuromelanin (*p < 0.05) and α-synuclein (*p < 0.05) of all combined regions in Phalaris-affected over unaffected EGK’s.

Due to the neurologic signs of Phalaris staggers in EGK and the presence of high concentrations of intra-neuronal neuromelanin, known to be associated with early Parkinsonian syndrome^39^, we investigated the presence of α-synuclein in the neurons and sera of all Phalaris-affected EGK via immunohistochemistry, immunofluorescence and ELISA. Initially, in the Phalaris-affected EGK brains we investigated the presence of the neuropathological hallmarks associated with human Parkinson’s disease (Fig. S2), including Lewy bodies and associated Lewy neurites^39^ following staining with the 97/8 rabbit anti-human α-synuclein antibody. Immunohistochemistry staining with 97/8 antibody revealed a fine punctate to prominent diffuse perikaryal pattern in the brain and spinal cord of Phalaris-affected EGK but failed to show presence of the Parkinson’s disease associated pathognomonic Lewy bodies and Lewy neurites (Fig. 4D–L). In order to confirm the specificity of the staining for α-synuclein, we also used the MJFR1 rabbit anti-human α-synuclein antibody raised against human recombinant full length α-synuclein and mapped to amino acids 118–123 (VDPDNE). MJFR1 antibody displayed dense and intense staining for α-synuclein in the brain and spinal cord of Phalaris-affected EGK (Fig. 4M–R). Immunofluorescence co-localisation studies confirmed homing of α-synuclein aggregates to neurons following double-staining with 97/8 and NeuN antibody (Fig. 5). Of note, the staining of the control unaffected EGK with 97/8 rabbit anti-human α-synuclein antibody only showed expression of endogenous α-synuclein homing to synaptic areas (data not shown). Finally, quantification of neuromelanin- and α-synuclein-laden neurons levels was perfumed using *imagej* analysis (Figs 6, S3). Levels of both neuromelanin and α-synuclein in the brains and spinal cords of Phalaris-affected EGKs were significantly higher compared to the unaffected EGKs (p < 0.05).

**Figure 7:**
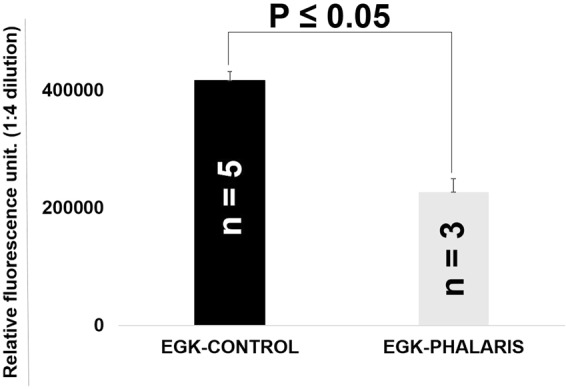
Immunodetection of Phalaris-associated α-synuclein by ELISA. Serum α-synuclein Average levels of α-synuclein in Phalaris-affected kangaroos (EGK89; EGK90 and EGK91; n = 3) were compared with average levels of α-synuclein in control healthy EGKC (n = 5). Error bars represent the mean level derived from n = 3 wells.

We then attempted to quantify levels of α-synuclein in sera derived from Phalaris-affected EGK by ELISA (Fig. 7). The immunoassay relied on the use of Syn-1 antibody for immunocapture and FL140 antibody to specifically immunodetect α-synuclein with no cross reactivity to β- and γ-synuclein. In 3 EGK tested, serum α-synuclein levels were shown to be significantly lower compared to the EGK controls (p < 0.05).

### Tryptamine alkaloids found in Phalaris spp were not detected in sera derived from kangaroos

Sera from Phalaris-affected EGK and control wallaby were used in an attempt to determine whether tryptamine alkaloids found in Phalaris grass can be detected via alkaloid liquid chromatography-electrospray ionisation mass spectrometry (LC-ESIMS). A selection of ten authentic tryptamines was obtained and their respective LC retention times and [M + H]^+^ parent ion masses (Table 1) were measured in positive ion mode, to aid analysis of the kangaroo samples. A first set of analytical experiments consisted of attempting to extract each of the target parent masses from the total ion chromatogram for each animal sample, run over the mass range *m/z* 150–600. Using this method, none of the standard tryptamines could be detected, except possibly for *N,N*-dimethyltryptamine (DMT) in samples derived from EGK and the wallaby. Although the parent ion corresponding DMT (*m/z* 189.14) was present in the EGK samples at the expected retention time of 4.97 minutes, further evidence was sought before the presence of this compound could be confirmed. A second set of experiments were conducted in which a data dependent acquisition (DDA) method was applied. The three most abundant ions within a duty cycle were selected for collision induced dissociation and their respective tandem mass spectra recorded. Fragment ions for each relevant MS/MS spectrum from EGK and wallaby samples were compared with those obtained experimentally from respective standards, each cross-referenced against published spectra^40^. This time none of the target tryptamines could be identified in any of the EGK samples. It is possible that the concentration of sample tryptamines was so low that they escaped detection using the DDA experiment. In an attempt to overcome such a possibility, a third and final set of experiments were conducted. These consisted of LC separation followed by targeted tandem mass spectrometry of three analytes: DMT (*m/z* 189.14, RT 4.97 min), 5-methoxy-*N,N*-dimethyltryptamine (*m/z* 219.15, RT 5.05 min) and bufotenine (*m/z* 205.13, RT 3.37 min). In each of the samples analysed, both 5-methoxy-*N,N*-dimethyltryptamine and bufotenine were clearly absent, as evidenced by a lack of match between the tandem mass spectra of standard and sample over an identical retention time window. The results for DMT are shown in Fig. S4. In the spectrum of authentic DMT (bottom), the parent ion is visible at *m/z* 189.15, with major fragment ions at *m/z* 144.09, 117.08, 91.06 and 58.07. It is clear that the spectrum from the wallaby sample lacks the parent ion, and hence can be discounted. EGK samples have spectra containing a parent ion at nominal mass 189, but the mass defect in each differs from the authentic significantly – casting serious doubt on the structure of the ion. Furthermore, despite the fact that the mass spectra of the EGK samples contain most of the fragment ions of authentic DMT, there is a conspicuous absence of *m/z* 58.07 for each. Thus, none of the samples contained DMT at a detectable concentration.

**Table 1:** Selection of ten authentic tryptamines alkaloids derived from Phalaris grass (provided by Dr Larry Walker).

Compound	Structure	Molecular formula	m/z of + ve parent ion (Th)	Retention time (mins)
Tyramine		C8H11NO	138.0919	ND
5-Methyltyramine		C9H13NO	152.1075	6.21
Tryptamine		C10H12N2	161.1079	5.04
Gramine		C11H14N2	175.1235	4.72
Hordenine		C10H15NO	166.1232	2.82, 3.06?
Bufotenine		C12H16N2O	205.1341	3.37
*N-N*-Dimethyltryptamine		C12H16N2	189.1392	4.97
5-Methoxy-*N,N-*dimethyltryptamine		C13H19N2O	219.1497	5.05
5-Methyltryptamine		C11H14N2	175.1235	Not actually part of the mixture!
5-Methoxytryptamine		C11H14N2O	191.1184	5.10
6-Methoxy-1,2,3,4-tetrahydro *β*-carboline		C12H14N2O	203.1184	5.40
2-Methyl-1,2,3,4-tetrahyro-*β*-carboline		C12H14N2	187.1235	5.68

## Discussion

In this study, we describe, and for the first time, cases of α-synucleino-neuromelanopathy in eastern grey kangaroos associated with Phalaris poisoning. The affected animals appear to display similar signs to those described by Bacci and colleagues affecting kangaroos^22^ and sheep^12,18,22,24,41^ and included ataxia, generalised muscle tremors, hyper-excitability, convulsions and head shaking as well as erratic hopping and flattened ears (in the case of kangaroos). Animals tended to display progressive gait abnormalities and pelvic limb paresis leading to collapse and death^18,26^. The above clinical presentation combined with the medical history and neuropathological findings together with the knowledge that kangaroos were grazing in a Phalaris-rich region during a climatic period that favoured the growth of alkaloid-rich Phalaris grass^12,18,22,24,41^ warranted the diagnosis of Phalaris staggers. In agreement with previous reports, the classical gross macroscopic greenish discoloration of the brain and spinal cord was observed; this presentation was exclusive to the CNS and was not observed in the kidneys of the affected kangaroos in contrast with previous reports^22^, indicating a more time-dependent chronic process allowing perhaps a faster and more efficient metabolism of the alkaloids. Furthermore, routine H&E stain revealed the presence of perikaryal melanin-like brown pigments affecting mainly the neurons of various areas of the brain and spinal cord. Moreover, we show that the neurons of the submucosal plexus of intestinal tract of the affected animals also contained melanin-like granules. Thus, a direct effect of the alkaloids on the ENS following ingestion of Phalaris grass can be hypothesized, possibly an early pathologic process/ event that precedes CNS pathologic changes.

Of note, an emerging concept in Parkinson’s disease research is the important role of the gastrointestinal tract in the pathogenesis of this disorder^42^. Braak and colleagues hypothesized that a prion-like agent undergoes retrograde transport^43^ from the ENS to the CNS via the vagus nerve of Parkinson’s disease patients lesions in the enteric nervous system precede those in the central nervous system^44^. Furthermore, Lebouvier and colleagues confirmed the presence of Parkinson’s disease pathologic changes in colon biopsies, further emphasizing the important role played by the gut in pathogenesis^45^. Despite the confirmation of the neuromelanin-like structure of the pigments in the neurons following staining with Warthin-Starry reaction and ultrastructural assessment with transmission electron microscopy, we were unable to conclusively identify any of the alkaloids normally found in Phalaris grass in the sera of the affected kangaroos using LC-ESIMS. Although the technique used to detect alkaloids in sera is highly sensitive, it is possible that these toxic compounds undergo fast metabolism and/or were not present at a detectable level in blood. Of note monoamine oxidase (MAO) leads to the breakdown of DMT into inactivated compounds while β-carbolines are potent inhibitors of the enzyme MAO and prevents DMT breakdown^46^, and both DMT and β-carbolines can be detected in blood following oral ingestion (20; 21; 85). Furthermore, both DMT and β-carbolines are found in Phalaris grass, so we speculate that the presence of β-carbolines in Phalaris grass led to accumulation of DMT following inhibition of MAO in kangaroos. It is also recognized that a number of neurotransmitters, including norepinephrine, epinephrine, dopamine and serotonin are degraded physiologically by MAO and lead to the production of deaminated metabolites detectable in urine^46^. It would be worthwhile conducting a separate LC-MS study in an attempt to detect either alkaloids via their metabolites in blood or the deaminated metabolites in urine of kangaroos affected with Phalaris staggers.

A significant finding reported here is the presence of α-synucleopathy in the brains and spinal cord of Phalaris-affected EGK. α-synuclein appeared as multi-shaped aggregates ranging from ovoid and fusiform to threadlike intensely stained structures. These presentations have been described previously by others and are believed to represent the aggregated neurotoxic species of α-synuclein^47–51^. Of note, α-synuclein was not restricted to the substantia nigra pars compacta but was widely distributed in several areas of the brain and spinal cord and affecting mainly morphologically normal neurons and closely associated with neuromelanin. This widespread expression might reflect an acute presentation following Phalaris poisoning akin to a stress response. Nevertheless, the ectopic perikaryal localization of α-synuclein, a cytoplasmic, presynaptic protein^52–55^, away from axon terminals, together with the abnormal multi-shaped deposits is potentially a causative effect of the alkaloids. The multi-location of abnormal α-synuclein in synucleinopathies is widely recognized and reviewed in^1^. An important report by Hirsch and colleagues demonstrated that neuronal loss of the midbrain dopamine-containing cell groups directly correlated with levels of neuromelanin in PD^56^. Halliday and colleagues demonstrated that pathologic change-free-morphologically-normal dopaminergic neurons (A9 neurons) in patients with early but definite Parkinson’s disease displayed higher levels of neuromelanin with its lipid component containing α-synuclein^39^; in contrast A9 neurons derived from late stage Parkinson’s disease with pronounced Lewy body pathologic change displayed significant loss of neuromelanin^39^. The Authors concluded that the changes observed were a prelude to the pathologic findings seen in later stages of the disorder^39^. The intracellular changes observed in the neurons of these kangaroos might reflect a similar progression but whether this is the case needs further confirmation. Finally, levels of α-synuclein aggregates were shown to be lower in sera derived from Phalaris-affected kangaroos when compared to healthy and unaffected EGK. Despite the inconsistency of the previously reported results linking plasma/serum α-synuclein levels with Parkinson’s disease severity/progression, it is generally accepted that these are decreased in this disorder^57^. Our results are consistent with previous studies that used blood samples derived from cases of Parkinsonism (reviewed in^58^).

In this report, we described cases of synucleinopathies in Eastern Grey Kangaroos following ingestion of neurotoxic, alkaloid-rich Phalaris. The kangaroos exhibited signs of a progressive neurologic disorder associated with neuropathologic changes that included accumulation of α-synuclein aggregates and neuromelanin without affecting the normal cell morphology. This study failed to demonstrate the presence of typical neuropathologic changes associated with Parkinson’s disease, namely Lewy bodies and Lewy neurites, probably reflecting an acute onset in the synucleinopathies spectrum. Further studies in kangaroos, following chronic poisoning with alkaloids, are needed to assess their long-term effects.

## Materials and Methods

### Humans, animals and ethics statement

#### Phalaris-affected kangaroos

Nine juvenile or adult Eastern Grey Kangaroos (EGKs, *Macropus giganteus*) (Table 2) were submitted for routine teaching/diagnostic post-mortem examination to UoM Veterinary Diagnostic Pathology and as such are not subject to animal ethics guidelines. All affected EGK animals were from various rural areas in the state of Victoria, Australia. All EGKs were grazing on Phalaris pastures in May 2016 when weather conditions were reported to favour rapidly growing young Phalaris grass (that is, a prolonged dry spell followed by heavy rain; Fig. S1).

**Table 2:** Clinical and epidemiological description of cases of kangaroos affected with Phalaris alkaloids and the Victoria regions and description of the unaffected control EGK from the NSW regions.

*Case No*.	*Year*	*Month*	*Location Victoria/NSW*	*Species*	*Age Category*	*Sex*	*Weight (Kg)*	Clinical Signs*
***PHALARIS AFFECTED EGK***								
EGK89	2016	May	Longlea, VIC	EGK*	Adult	Male	12.35	Ataxia
EGK90	2016	May	Taradale, VIC	EGK	Adult	Female	15	Ataxia, ears flaccid, head shaking, ‘pogo stick’ hopping
EGK91	2016	May	Taradale, VIC	EGK	Sub Adult	Female	12	Ataxia, erratic hopping
EGK92	2016	May	Taradale, VIC	EGK	Sub Adult	Male	10.8	Ataxia, erratic hopping
EGK46	2016	May	Sedgwick, VIC	EGK	Adult	Female	15	Ataxia
EGK59	2016	May	Springfield, VIC	EGK	Adult	Female	15	Ataxia, ears flaccid
EGK53	2016	May	Victoria, VIC	EGK	Adult	NR	NR	Ataxia
EGK42	2016	May	Victoria, VIC	EGK	Adult	NR	NR	Ataxia
***UNAFFECTED EGK AND SWAMP WALLABY***								
SW47	2016	May	Portland, VIC	Swamp Wallaby (control)	Adult	NR		Weakness
EGKC1	2018	July	Colyton, NSW	EGK	Young	Male	4	None
EGKC2	2018	July	Colyton, NSW	EGK	Young	Female	4	None
EGKC3	2018	July	Camden, NSW	EGK	Young	Male	5	None
EGKC4	2018	July	Blacktown, NSW	EGK	Young	Female	10	None
EGKC5	2018	July	Plumpton, NSW	EGK	Young	Male	8	None

#### Unaffected kangaroos

Ten juvenile or adult Eastern Grey Kangaroos (EGKs, *Macropus giganteus*) (Table 2) were submitted for routine clinical examination to the Sydney Metropolitan Wildlife Services (sera; n = 5) and for routine diagnostic post-mortem examination to the New South Wales Department of Primary Industries (brains; n = 5) and as such are not subject to animal ethics guidelines. Sera derived from blood samples taken from the lateral tail vein of five healthy unaffected EGK (Table 2), were used to compare the levels of α-synuclein aggregates. Brain sections derived from five healthy unaffected EGK (Table 2), were also used. All unaffected EGK animals were from various rural areas in the state of NSW, Australia.

#### Parkinson’s disease and control human samples

The human Parkinson’s disease and control brain samples were provided by the Victorian Brain Bank which operates under the jurisdiction of the Human Research Ethics Committee of UoM, Ethics ID: 1545740 - Victorian Brain Bank - Brain banking for neuroscience research and The Victorian Institute of Forensic Medicine Ethics ID: EC12/2016 - Victorian Brain Bank (VBB). Brain Banking for Neuroscience Research – Studies on Neurological and Psychiatric illness. All samples where de-identified and coded prior to handling.

### Clinical investigations

All the EGK found in Victoria displayed signs of ataxia and the majority exhibited other neurological signs including erratic hopping, flaccid ears, generalised muscle tremors and head shaking. The animals displayed signs of hyperexcitability when frightened and tended to hop erratically with complete failure to coordinate movements leading to falls and inability to stand. All animals were submitted for diagnostic investigation, including macroscopic and histological assessments.

### Histopathology and immunohistochemistry

The brains of the EGK and wallaby found in Victoria were sectioned into two halves; the left half was frozen at −80 °C and the right half was fixed by immersion in 10% neutral buffered formalin. Tissue slices from different anatomical regions of the cerebrum, midbrain and cerebellum as well as the intestinal tract were then processed through graded alcohols and xylene and embedded in paraffin. The tissues were sectioned at 4 µm for staining with haematoxylin and eosin (H&E), Warthin-Starry, Fontana-Masson, Shmorl’s or immunohistochemistry (IHC) using anti-α-synuclein antibody. Staining for α-synuclein was performed as described^59^. Serial sections were first deparaffinised then treated with 80% formic acid for 5 min. This was followed by adding 3% hydrogen peroxide to block endogenous peroxidase activity. Sections were then stained with the 97/8^59^ or MJRF1 (abcam, ab138501) rabbit anti-human α-synuclein polyclonal IgG [97/8; 1:2000 dilution], for 1 h at room temperature. Visualization of antibody binding was achieved using the LSAB™ kit (labelled streptavidin–biotin, DAKO) and sections were then incubated with hydrogen-peroxidase–diaminobenzidine (H_2_O_2_–DAB) to visualize α-synuclein. Sections were counterstained with Mayer’s haematoxylin. Sections were then viewed using a light microscope (Olympus BH-2) and scanned using a Zeiss Mirax Digital Slide Scanner (Zeiss). Digital images were taken from e-slides using the Pannoramic Viewer software analysis (3DHISTECH).

### Image quantification

To quantify neurons displaying accumulation of neuromelanin or α-synuclein, signal intensity was visualised by capturing field images by Olympus VS 120 Slide Scanner and images were analysed by ‘Olympus OlyVIA’ software. Neuromelanin- or α-synuclein-laden neurons were quantified by an image processing software, Image J. The mean colour threshold of ‘particles’ was calculated in several brain regions [cerebrum (=5), midbrain (n = 5) and cerebellum (n = 5)] and spinal cord (n = 5) of each Phalaris-affected and unaffected kangaroos obtained with a 40 × objective, numerical aperture 0.95. The final result was presented as percentage intensity and expressed as mean ± S.E.M.

### Immunofluorescence staining and imaging of α-synuclein aggregates and NeuN

Brain sections were processed as described above prior to staining with rabbit anti-human α-synuclein polyclonal IgG [97/8; 1:2000 dilution] or anti-mouse NeuN IgG monoclonal antibody (Millipore, 1:2000 dilution), for 1 h at room temperature. After washing, this was followed by the secondary antibody diluted in PBS [(anti-rabbit IgG whole molecule FITC-conjugate produced in goat, Sigma and anti-mouse IgG (H + L) Texas-red-conjugate produced in Donkey, Sigma respectively)] for 1 hour at RT.

After the final wash in PBS, fluorescence anti-fade solution (Invitrogen) was added to slides and coverslips were mounted and sealed with clear nail polish to prevent dehydration. Florescence microscopy was performed with a Leica DM4000B microscope. Images from each source [FITC (450–490 nm), and Texas red (510–560 nm)] were collected by a high resolution DC500 colour camera attached. All images are saved digitally using Leica’s IM500 Image Manager Database software from the same field-of-view. Images were merged using Photoshop 6.0 (Adobe). Confocal laser scanning microscopy was performed with a Zeiss LSM510 confocal system on an inverted Zeiss Axio100M. Z-series and snapshot images were collected. Dual scans were merged using Photoshop 6.0 (Adobe).

### Electron microscopy

The formalin-fixed cerebral cortex derived from a Phalaris-affected EGK and the control wallaby were fixed in 2.5% glutaraldehyde (ProSciTech, Australia) then washed in phosphate buffer and post fixed in 1% osmium tetroxide (ProSciTech, Australia). The specimens were dehydrated in acetone and embedded in Spurr’s resin (ProSciTech, Australia). One-micron semi-thin sections were stained with methylene blue for light microscopy. Ultrathin sections were double stained with uranyl acetate and Reynold’s lead citrate and examined with a Philips CM10 transmission electron microscope at 60 kV.

### Sandwich ELISA for the detection of α-synuclein in sera of Phalaris-affected kangaroos

The assay was performed as described with slight adaptation^60^. A 384 well plate (Black, Maxisorp; Nunc) was coated with an IgG1 mouse anti-α-synuclein monoclonal antibody (0.25 μg/ml); Syn-1 (BD Bioscience) in 200 mM NaHCO3 coating buffer, pH 9.6 (50 μl/well) overnight at 4 °C. The plates were then washed four times with PBST (PBS containing 0.05% Tween-20) and subsequently blocked with 100 μl/well of gelatine blocking buffer (PBS containing 2.25% gelatine and 0.05% Tween-20) for 2 hr. at 37 °C. The plates were then washed four times with PBST. 50 μL of serum samples (1:4 dilution) derived from the Phalaris-affected (Victoria) and the unaffected (NSW) EGK as well as standards diluted in signal boost solution 1 (Signal Boost Immunoreaction Enhancer Kit, Merck) were then added to each well, and incubated overnight at 4 °C. The plates were then washed four times with PBST followed by the addition of 50μl/well of IgG anti-α/β/γ-synuclein rabbit polyclonal antibody (FL-140) (Santa Cruz Biotechnology, diluted to 1:1000 in Signal Boost solution 1) and incubated at 37 °C for 1.5 hours. The plates were then washed four times, then incubated for 1 hour at 37 °C with 50 μl/well with goat anti-rabbit horseradish peroxidase (Jackson Immunoresearch) diluted 1:10000 in Signal boost solution 2 (Merck). Finally, plates were washed again and 50 μl/well of an enhanced chemiluminescent substrate (Super-Signal ELISA Femto, Pierce Biotechnology) was added. Chemiluminescence in relative light units was immediately measured with a Victor3 1420 (Wallac) microplate reader at 470 nm. Purified recombinant α-synuclein (Monash University protein unit) was used as standards and samples were assayed in duplicate.

### Mass spectrometry

Phalaris alkaloid standards (Table 1) were sourced from Southern Scientific, Hamilton, Victoria, Australia and included tyramine, tryptamine, gramine, hordenine, bufotenine, *N,N*-dimethyltryptamine, 5-methyltyramine, 5-methoxytryptamine, 5-methoxy-*N,N*-dimethyltryptamine, 6-methoxy-1,2,3,4-tetrahydro-*β*-carboline, and 2-methyl-1,2,3,4-tetrahydro-*β*-carboline in methanolic solution. Dilution in LC-MS grade acetonitrile (Burdick and Jackson) to approximately 10 ppm component concentration was performed prior to LC-MS analysis. 20 µL of each serum sample derived from Phalaris-affected EGK and control wallaby was added to 60 µL LC-MS grade acetonitrile to precipitate total protein content. Following centrifugation of the solution at 14,000 rpm for 10 min, the supernatant was carefully placed in a Waters Total Recovery chromatography sample vial for analysis.

Liquid chromatography was performed using a Waters Acquity UPLC equipped with a Waters Acquity C18 HSS column of dimensions 2.1 × 150 mm, thermostatted to 35 °C. Solvent A consisted of ultrapure water (Milli-Q) plus 0.1% formic acid and solvent B consisted of LC-MS grade methanol (Burdick and Jackson) plus 0.1% formic acid. A 20 min run was employed with a flowrate of 0.20 mL/min. An initial solvent composition of 5% B was ramped linearly to 100% B over 10 minutes. After 15 minutes the solvent composition was immediately returned to 5% B and maintained until the end of the run. Injections of 5 µL were made from sample solutions stored at 4 °C.

Mass spectrometry was performed using a Waters Xevo QToF-MS spectrometer fitted with an electrospray ionisation probe and operating in positive ion mode. Mass accuracy was maintained by infusing at 5 µL/min a lock spray solution of 200 pg/µL leucine encephalin in 50% aqueous acetonitrile, plus 0.1% formic acid, calibrated against a sodium iodide solution. The capillary voltage was maintained at 2.3 kV, cone voltage at 25 V, desolvation temperature 350 °C, ion block temperature 120 °C, gas (N_2_) flows: desolvation 450 L/hr., cone gas 10 L/hr. MassLynx software (Waters) was used to process the data. Three different types of LC-MS experiments were performed: (i) a simple acquisition over the mass range *m/z* 150–600, scan time 1 s; (ii) a targeted MS/MS of three analytes: *m/z* 205.1 (bufotenine), *m/z* 219.1 (5-methoxydimethyltryptamine), and *m/z* 189.1 (dimethyltryptamine), each with a scan time of 0.5 s. Collision energy was ramped from 10–40 eV and MS/MS spectra were recorded over the range *m/z* 50–600; and a data dependent acquisition (DDA) experiment. Parent ion survey (1 s scan time) with mass range *m/z* 150–600, and fragment ion mass range *m/z* 50–600. Singly charged ions above 50 cps intensity were fragmented for 2 s with a collision energy range of 10–35 eV (low mass) to 15–40 eV (high mass). A total of 3 ions were selected within a given scan, and after fragmentation excluded from detection for 30 s.

### Statistical analysis

One-way ANOVA with Dunnett’s post-test was performed using GraphPad Prism version 7.00 for Windows, GraphPad Software, San Diego California USA, for statistical analysis.

## Supplementary information

Supplementary file
