# Supplementary material for: Plant poisoning leads to alpha-synucleinopathy and neuromelanopathy in kangaroos
Source: Sci Rep. 2019 Nov 13;9:16546. doi: 10.1038/s41598-019-53396-8 (PMC6853926; doi:10.1038/s41598-019-53396-8)
Supplement: Supplementary file 1 — Supplementary file [file 41598_2019_53396_MOESM1_ESM.pdf]

Plant poisoning leads to alpha-synucleinopathy and neuromelanopathy in kangaroos

Mourad Tayebi[1,4]* Charles M El-Hage[2], Pedro Pinczowski[3], Pam Whiteley[2], Monique David[1], Qiao-Xin Li[3], Shiji Varghese[4], Meena Mikhael[1], Umma Habiba[1], David Harman[1], Liliana Tatarczuch[2], Mirjana Bogeski[4], Ian Birchall[4], Kirsty Ferguson[5,6], Larry Walker[7], Colin Masters[4], Brian A. Summers[8].

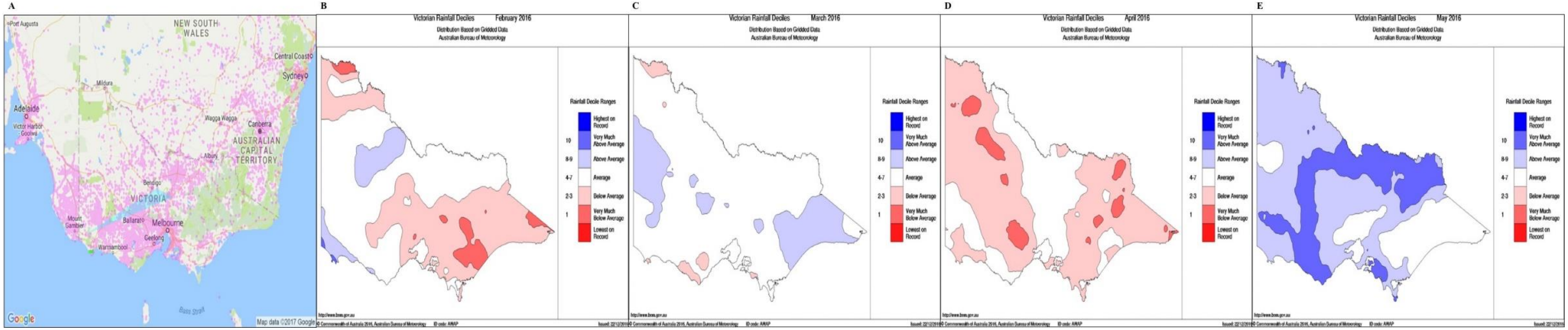

Figure S1: (a) Phalaris grass distribution in the Victorian region. Derived from Google Map. (b) Weather pattern on the months preceding ingestion with Phalaris grass. Information about weather patterns can be accessed on the following links for:
February 2016 (http://www.bom.gov.au/jsp/awap/rain/archive.jsp?colour=colour&map=decile&year=2016&month=2&period=month&area=vc);
March 2016 (http://www.bom.gov.au/jsp/awap/rain/archive.jsp?colour=colour&map=decile&year=2016&month=3&period=month&area=vc);
April 2016 (http://www.bom.gov.au/jsp/awap/rain/archive.jsp?colour=colour&map=decile&year=2016&month=4&period=month&area=vc);
and May 2016 (http://www.bom.gov.au/jsp/awap/rain/archive.jsp?colour=colour&map=decile&year=2016&month=5&period=month&area=vc) expressed in Rainfall Deciles. Derived from the Australian Bureau of Meteorology, National Climate Centre according to licence terms (https://creativecommons.org/licenses/by/3.0/au/). No modifications or changes have been made to the original weather maps.

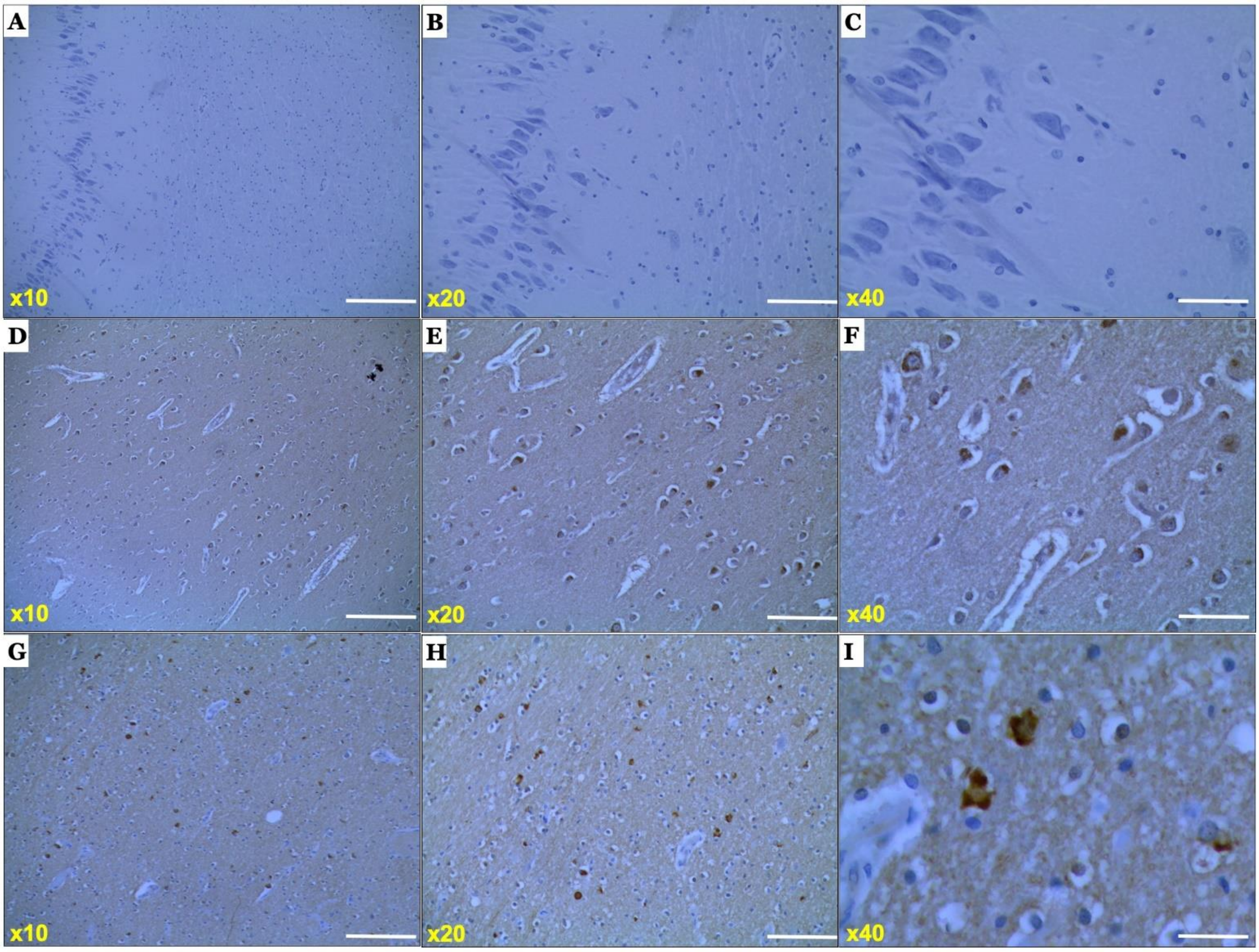

Figure S2: Photomicrographs of the alpha-synucleinopathy in the central nervous system derived from human Alzheimer (a) Immunohistochemical staining with secondary antibody (no primary control of a human Parkinson's disease brain. b) and c) are higher magnification of a). (d) Immunohistochemical staining with 97/8 rabbit anti-human alpha-synuclein polyclonal IgG [1:2000 dilution] of a human Parkinson's disease brain with typical alpha-synucleinopathy lesions. e) and f) are higher magnification of a). g) Immunohistochemical staining with anti-human synuclein MJRF1 rabbit anti-human alpha-synuclein polyclonal IgG [1:2000 dilution] antibody of a Parkinson's disease brain. (h) and i) are higher magnification of g).

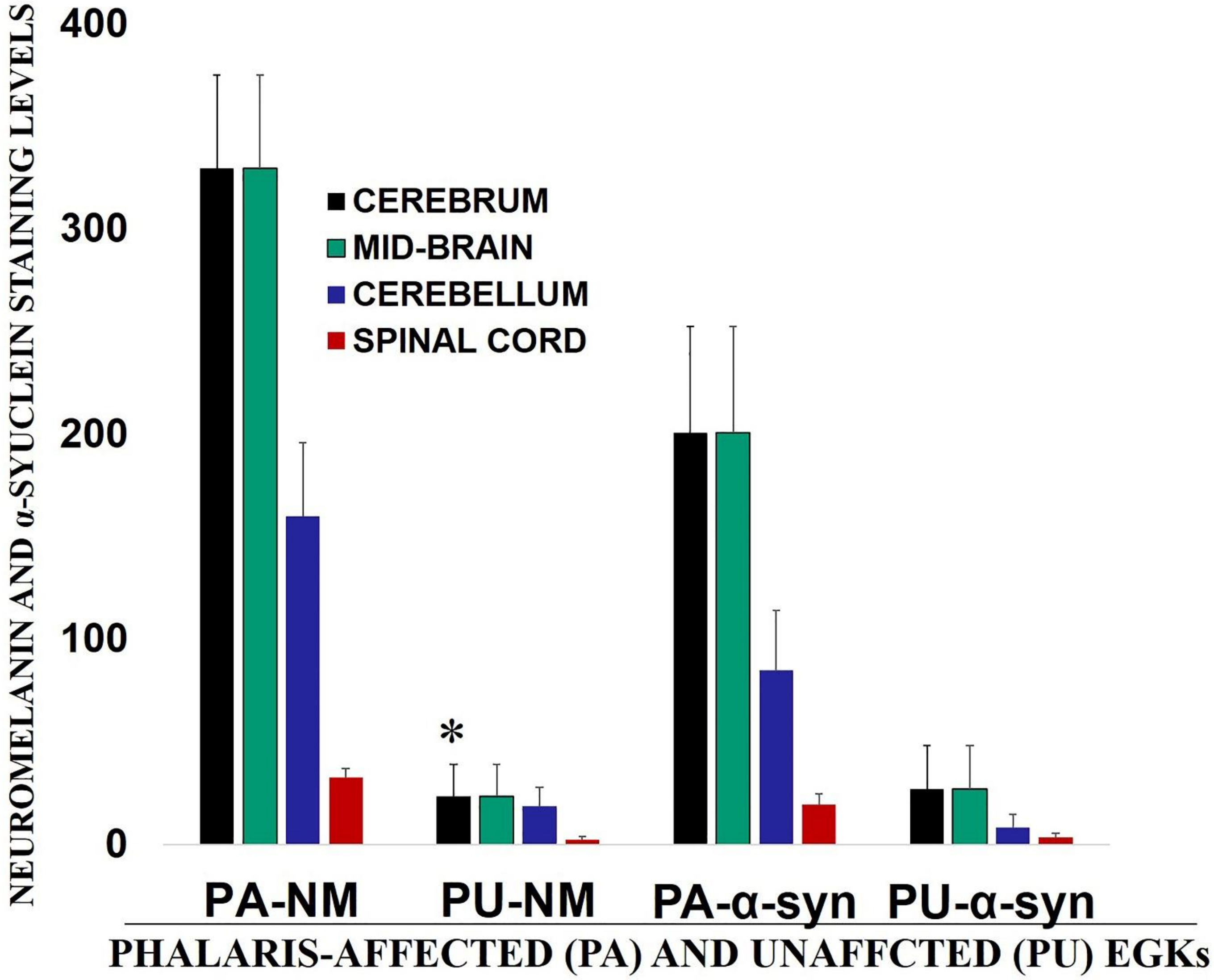

Figure S3. Quantitation of neuromelanin- and alpha-synuclein-laden neurons in cerebrum, mid-brain, cerebellum and spinal cord regions. Neuromelanin and alpha-synuclein staining levels of Phalaris-affected (n =8) EGKs. The data are mean ± SEM from 5 randomly selected images per section. Similar regions from unaffected (n = 4) EGKs were used as a control. Neuromelanin and alpha-synuclein staining levels were quantified via ImageJ software as the relative intensity of neuromelanin (*p < 0.05) and alpha-synuclein (*p < 0.05) of cerebrum, mid-brain, cerebellum and spinal cord regions in Phalaris-affected over unaffected EGK's.

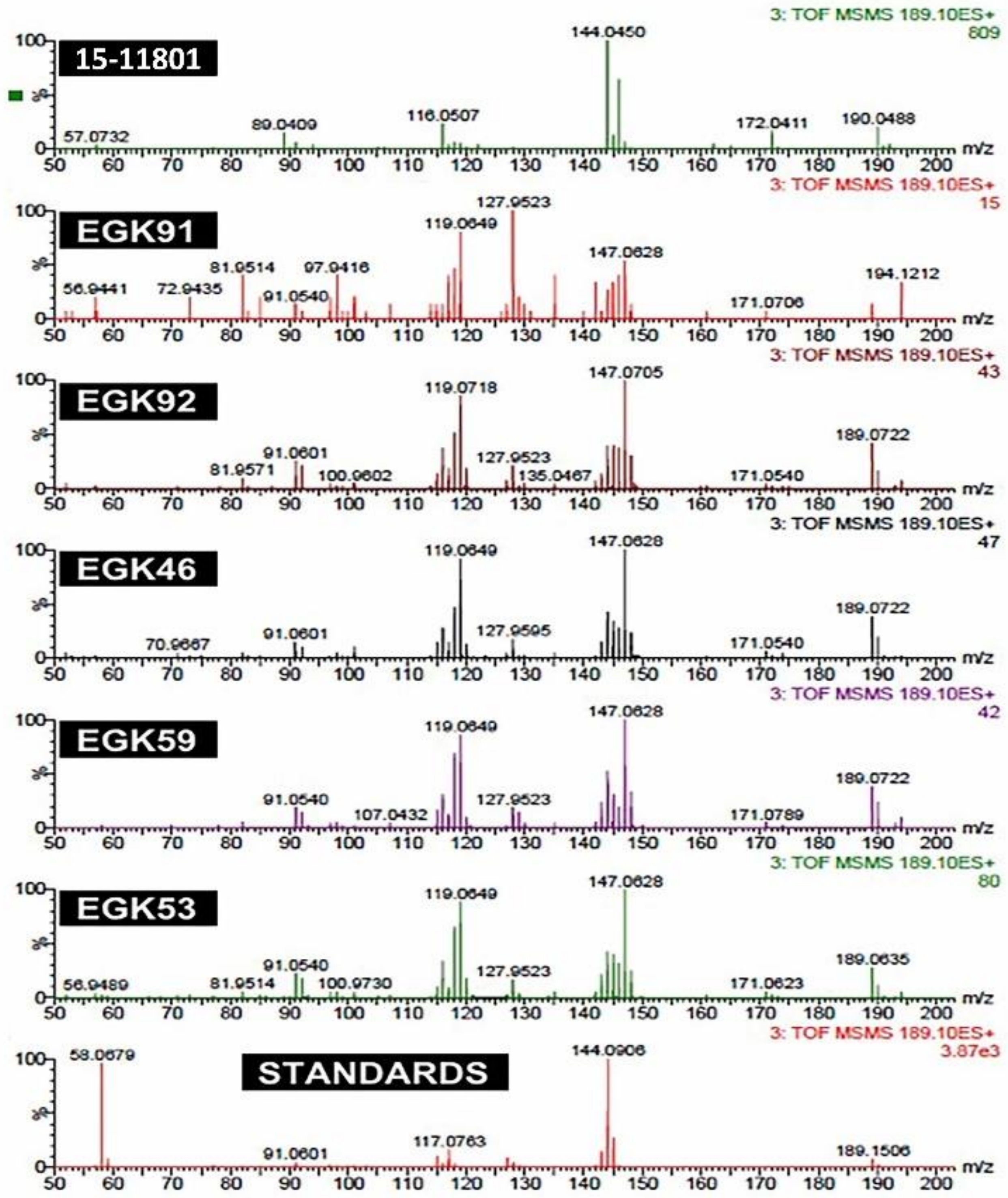

Figure S4: Liquid chromatography-electrospray ionisation mass spectrometry assessment of sera derived from Phalaris-affected EGK and control wallaby.
